# The Influence of Shc Proteins and Aging on Whole Body Energy Expenditure and Substrate Utilization in Mice

**DOI:** 10.1371/journal.pone.0048790

**Published:** 2012-11-07

**Authors:** Jennifer H. Stern, Kyoungmi Kim, Jon J. Ramsey

**Affiliations:** 1 VM Molecular Biosciences, University of California Davis, Davis, California, United States of America; 2 Department of Public Health Sciences, University of California Davis, Davis, California, United States of America; Institut Pluridisciplinaire Hubert Curien, France

## Abstract

While it has been proposed that Shc family of adaptor proteins may influence aging by regulating insulin signaling and energy metabolism, the overall impact of Shc proteins on whole body energy metabolism has yet to be elucidated. Thus, the purpose of this study was to determine the influence of Shc proteins and aging on whole body energy metabolism in a mouse model under ambient conditions (22°C) and acute cold exposure (12°C for 24 hours). Using indirect respiration calorimetry, we investigated the impact of Shc proteins and aging on EE and substrate utilization (RQ) in p66 Shc−/− (ShcKO) and wild-type (WT) mice. Calorimetry measurements were completed in 3, 15, and 27 mo mice at 22°C and 12°C. At both temperatures and when analyzed across all age groups, ShcKO mice demonstrated lower 24 h total EE values than that of WT mice when EE data was expressed as either kJ per mouse, or adjusted by body weight or crude organ mass (ORGAN) (P≤0.01 for all). The ShcKO mice also had higher (P<0.05) fed state RQ values than WT animals at 22°C, consistent with an increase in glucose utilization. However, Shc proteins did not influence age-related changes in energy expenditure or RQ. Age had a significant impact on EE at 22°C, regardless of how EE data was expressed (P<0.05), demonstrating a pattern of increase in EE from age 3 to 15 mo, followed by a decrease in EE at 27 mo. These results indicate a decline in whole body EE with advanced age in mice, independent of changes in body weight (BW) or fat free mass (FFM). The results of this study indicate that both Shc proteins and aging should be considered as factors that influence energy expenditure in mice.

## Introduction

The aging process is dependent on a combination of genetic and environmental factors. Understanding this relationship at both the cellular and whole animal level is a central challenge in studying the mechanisms that contribute to age-related dysfunction and pathology. Recently, several signaling molecules proposed to play a role in the aging process have been identified (i.e., molecules involved in the insulin/IGF1 signaling pathway, SIRT1, and the metabolic sensor AMPK) [1], [2]. The signaling molecule p66 Shc has also been reported to play a role in aging [3]. Three splice variants (p46 Shc, p52 Shc, and p66 Shc) are encoded by the mammalian Shc locus. The p66 Shc(−/−) mouse has been a common model used to investigate the possible link between p66 Shc and aging, however, it has recently been shown [4] that the levels of both the p52 Shc and p46 Shc isoforms are also substantially decreased in liver and skeletal muscle from these animals. Thus, these mice (we refer to as ShcKO) provide a model of overall decreases in Shc protein levels in muscle, liver and other tissues. Since the initial report linking Shc proteins to aging, numerous studies have attempted to identify the mechanism by which Shc influences aging [5]–[7]. While these studies suggest that Shc proteins may impact aging primarily by modulating mitochondrial ROS production and apoptosis, there is accumulating evidence that Shc proteins may also play a role in regulating energy metabolism. It has been reported that ShcKO mice resist weight gain on a high fat diet [4], [8] and decreased Shc levels in leptin-deficient Ob/Ob mice leads to an attenuation of weight gain and insulin resistance [9]. Thus, it is possible that alterations in energy metabolism may represent a fundamental mechanism by which Shc deficiency impacts healthy aging.

Shc proteins play a role in insulin signaling [10], [11] and recent evidence suggests that Shc proteins may influence aging through alterations in insulin signaling, adiposity, and energy metabolism [4], [8]. There is some indirect evidence suggesting that energy expenditure may be increased in ShcKO mice. It has been reported that body weights are lower in ShcKO compared to wild-type (WT) mice when consuming either a standard or high fat diet despite the fact that energy intake is not different between genotypes [4], [8]. Similarly, decreased Shc protein levels in leptin-deficient Ob/Ob mice leads to a decrease in weight gain without altering food intake [9]. To our knowledge, only one study thus far has measured whole body energy expenditure in ShcKO mice and this study reported that oxygen consumption (ml/g body weight) is increased in these animals compared to wild-type mice [8]. The results of these studies indicate that decreased Shc protein levels may mitigate weight gain by increasing energy expenditure. Thus, it is possible that decreased Shc protein levels may, in fact, stimulate whole body energy expenditure and/or attenuate any possible age-related decline in energy expenditure. In contrast to these studies, it has been reported that p66 Shc localizes to mitochondria and increases oxygen consumption [12], suggesting that oxygen consumption/energy expenditure may be decreased in ShcKO animals. It has also been reported that body temperature is decreased in ShcKO mice compared to wild-type animals following acute cold exposure, suggesting that ShcKO mice may have an impaired ability to increase energy expenditure [8]. Thus, the overall influence of Shc proteins on whole energy expenditure is still not entirely clear.

The purpose of this study was two-fold. First, we set out to determine if the energetic response to aging and acute cold exposure is altered in ShcKO mice. Second, we wanted to investigate the influence of aging and cold exposure on energy metabolism in mice. Relatively little is known about the influence of aging on whole body energy expenditure in mice, despite the fact that mice are a major model used for aging studies. In addition to measuring energy expenditure under typical ambient conditions (22°C), we also wished to determine the influence of Shc proteins on physiological response to an environmental condition (cold exposure) which stimulates energy expenditure. It has been documented that aging is associated with a diminished cold-induced increase in oxygen consumption and energy expenditure in mice [13]–[16], as well as humans [17]–[19]. In mice, these oxygen consumption/energy expenditure measurements are often completed in animals studied in environments very different from the home cage (i.e., lack of bedding, restraint) and exposed to temperatures ≤10°C [13]–[16]. Relatively little is known about the influence of aging (and Shc proteins) on the acute stimulation of energy expenditure in response to moderate cold (12°C) exposure in animals housed in an environment similar to the home cage.The purpose of this study was to investigate the impact of aging and acute cold exposure on whole animal energy expenditure and substrate oxidation in ShcKO and WT mice.

## Materials and Methods

### Ethics Statement

The animal use protocol was approved by the University of California – Davis Institutional Animal Care and Use Committee (Animal Welfare Assurance Number A3433–01). The study was conducted in accordance with the recommendations in the National Research Council Guide for the Care and Use of Laboratory Animals.

### Animals, Diet, and Energy Intake

ShcKO mice (C57Bl/6) were provided by Dr. Pier Giuseppe Pelicci (Department of Experimental Oncology, European Institute of Oncology, Milan, Italy) and used to establish a breeding colony at UC Davis. All mice in this study were on a C57/B6 background and have been previously described [3]. Heterozygous ShcKO mice were mated to produce founders for the lines of ShcKO and wild-type (WT) animals used in the present study. Prior to collection of indirect respiration calorimetry data, food intake and body weight was monitored for 7 days in weight stable 3 mo (n = 8 per genotype), 15 mo (n = 6 and 3 for ShcKO and WT, respectively), and 27 mo (n = 9 per genotype) male WT and ShcKO mice. Animals were individually housed in a light (12-h light/12-h dark cycle, lights on at 7 am, lights off at 7 pm) and temperature (22°C) controlled vivarium at the University of California-Davis (UCD). This study was approved by the UCD Animal Care and Use Committee. All mice were fed a commercial diet with an energy desity of 13.0 kJ/g) (7012 Teklad LM-485 Mouse/Rat Sterilizable Diet, Harlan USA; 25% protein, 17% fat, and 58% carbohydrate on a metabolizable energy basis). Only male mice were used for the present study and this reflects the fact that our initial studies investigating the influence of ShcKO on energy metabolism have been completed in male mice [20], [21]. Our goal was to initially use male animals to screen for changes in energy metabolism in the ShcKO animals. Future studies are needed in female mice to more completely determine the overall influence of Shc proteins on energy metabolism.

Ad libitum food intake was measured by weighing the amount of food remaining in the hopper at the same time daily, while accounting for any spillage by sifting bedding and weighing any food particles remaining in the cages. The calculated metabolizable energy (ME) of the diet (14.27 kJ/g) was used to determine metabolizable energy intake (MEI).

### Indirect Respiration Calorimetry

Total daily EE was measured using whole-body indirect respiration calorimetry. Prior to calorimetry measurements, all animals were adapted to the chambers for a period of 24 h at which time food intake was monitored to ensure that these values did not differ from previously collected data during adaptation to individual housing. Calorimetry measurements were completed for each animal on two individual 24 h data collection periods; an initial 24 h period at 22°C and a subsequent 24 h period under 12°C conditions. Each 24 h calorimetry data collection period began at approximately 10∶00 AM. Chambers had the same dimensions and shape as the animals’ home cage (Paige Instruments, Woodland, CA). Room air was drawn through the chambers at 400 mL/min. This flow rate was controlled and measured with a mass flow controller (MFS-5, Sable Systems International, Las Vegas, NV). Samples of room and chamber air were dried by a Peltier condenser (PC-4, Sable Systems) before entering Oxygen and CO_2_ analyzers. Oxygen content was measured by a fuel cell oxygen analyzer (FC-10, Sable Systems) and CO_2_ content was measured by an infrared CO_2_ analyzer (CA-10, Sable Systems). Calorimeter calibration was performed daily prior to beginning each 24 h measurement. A 1.9% CO_2_ reference gas, 100% Nitrogen gas, and dry room air were used to calibrate CO_2_ and Oxygen analyzers. Data from the mass flow controllers and gas analyzers were collected using a data acquisition system (UI2, Sable systems) with a PC using Expedata software (Version 1.3.0.12, Sable Systems). EE was calculated using the following modified Weir equation [22].
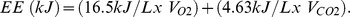



RQ was calculated as the ratio of volume of CO_2_ produced to the volume of O_2_ consumed. A food quotient of 0.87 was calculated from the proportions of protein, fat, and carbohydrates in the diet. The ratio of dark EE to light EE (D:L) was used to indicate the magnitude of diurnal changes in EE.

### Feeding Schedule

The animals were allowed access to food from 9 PM to 10 AM each day. At this time, food was pulled from cages and weighed and bedding was replaced to account for any food spillage that may have occurred during the feeding period. This allowed us to control periods of feeding and fasting and collect light cycle data which primarily reflected fasting RQ and energy expenditure. Thus, the light cycle measurements may more closely reflect resting conditions since these measurements are not interrupted with periods feeding and activity related to feeding. The mice were adapted to this feeding regimen for one week prior to the start of calorimetry measurements.

### Organ Weights and Body Composition

Immediately after collection of calorimetry data, animals were sacrificed via CO_2_ inhalation and cervical dislocation. Immediately following euthanasia, organs were collected, weighed, and returned to the carcass at which time the carcass was weighed and stored at −20°C for preparation of total body water (TBW) analysis. Carcasses were then freeze- dried for 7 days (until weight stable) to remove all fluids (Virtis Sublimator). Total body water was calculated by subtracting the freeze dried weight from the carcass weight. Fat free mass was determined by the following equation previously described [23], [24] [25]–[30].

Fat free mass = TBW/0.73.

### Statistical Analyses

Analysis of variance (ANOVA) was performed to determine if each of MEI, BW, FFM, ORGAN, EE, RQ, and D:L differed between genotypes at baseline 22°C conditions. Differences in organ weights and FFM were determined using ANOVA with linear random-effects models. EE is expressed as kJ/min/mouse (kJ per min per mouse), kJ/g BW/min (kJ per gram BW per min), kJ/min using BW as a covariate, and kJ/min using FFM as a covariate in the model. RQ is expressed as a raw value and as RQ adjusted for MEI (RQ_MEI_) as a covariate in the model. MEI from the previous feeding period was used to adjust fasting RQ. Analyses were performed separately by fasted/fed treatments which corresponded with light/dark cycle, respectively. To investigate possible differences in the energetic response to cold stress, individual trajectories of changes in EE, RQ, MEI, and D:L were compared between genotypes and ages by repeated measures analysis of variances (ANOVAs) using linear random-effects models. Each response level was entered as the dependent variable. The main effects of genotype, age, and temperature, and the interaction terms of genotype*age, genotype*temperature, age*temperature, and genotype*age*temperature were modeled as independent variables. To account for between subject heterogeneity in the changes of response levels, intercept and temperature were modeled as random effects. Multiple comparisons were controlled by the Bonferroni correction method where appropriate. Stepwise backward elimination process was performed to select the final model. We used 5% as the cut-off for the probability of dropping a variable from the full model. The probabilities were calculated using the Wald test and likelihood ratio test was performed to compare the initial model with the final model. Significance was defined as a two-sided *P*<0.05. All statistical analyses were performed using SAS version 9.3 (SAS Institute, Inc).

## Results

### Energy Intake

**Figure 1:**
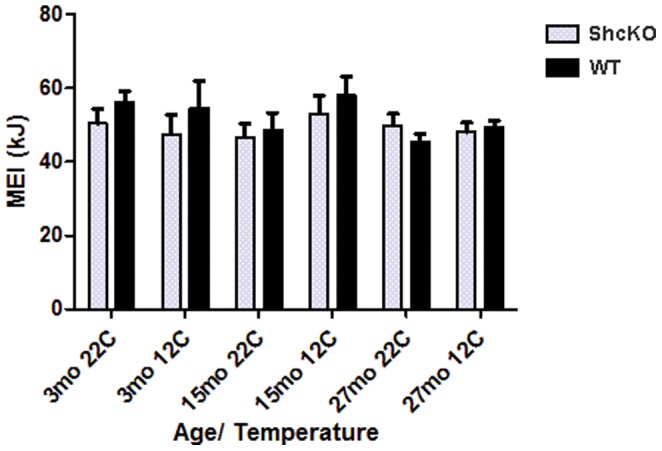
Metabolizable energy intake (MEI) in ShcKO and wild-type (WT) mice housed at 22°C or 12°C. Mean (±SEM) 24 hour energy intake measured in 3, 15 and 27 month old animals. Energy intake measurements at 22°C were completed over a 1 week period while 12°C measurements were completed during 24 hour acute cold exposure. ShcKO = p66 Shc(−/−) mice, mo = months.

Neither age, nor genotype had a significant main effect on energy intake at 22°C or 12°C. Repeated measures ANOVA also revealed that acute exposure to 12°C did not induce an increase in MEI in either genotype (Figure 1).

### Body Weight, Organ Weights, and Fat Free Mass

**Figure 2:**
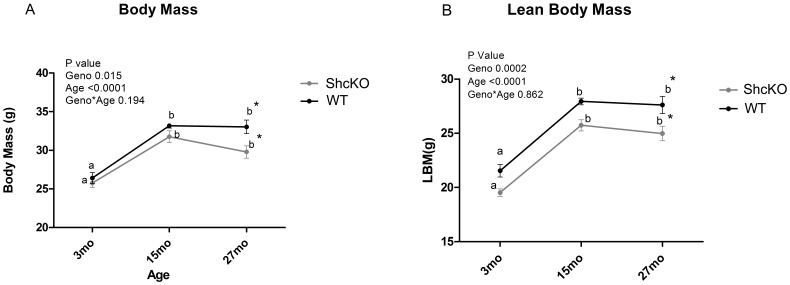
Body weight and fat free mass in ShcKO and wild-type (WT) mice. Mean (±SEM) body weight (A) and fat free mass measured in 3, 15 and 27 month old animals housed at 22°C. Letters that differ indicate significance within genotype between age, ANOVA bonferroni corrected P<0.0001; *Difference between genotypes within age P<0.05. ShcKO = p66Shc(−/−) mice, Geno = genotype, mo = months.

We found no evidence of a genotype*age interaction on either FFM or BW. That is to say, the two genotypes showed no differences in pattern of change in either FFM or BW with aging (Figure 2). In both genotypes BW and FFM demonstrated an age-related increase in mass from 3 to 15 mo. However, there was no significant change in either BW or FFM from 15 to 27 mo of age. There were no differences between genotypes in BW or FFM at either 3 or 15 months of age, although BW and FFM were decreased in the ShcKO compared to WT mice at 27 mo of age. We found a significant effect of age on the weights of all organs, with the exception of spleen. Similar to the age effect seen in BW and FFM, the impact of age on organ mass was consistently seen as an increase in mass from 3 to 15 mo of age and no significant change from 15 to 27 mo of age. Crude organ weight also demonstrated this same pattern of a significant age effect and, though not statistically significant, a trend of genotype effect (*P* = 0.071) with ShcKO animals having reduced crude organ weights compared to WT mice. This trend is primarily due to smaller liver weights among all ages (*P* = 0.070) of ShcKO compared to WT animals (Table 1).

**Table 1:** Organ weights and body composition in ShcKO and wild-type (WT) mice.^1^

	ShcKO			WT			*P* Value		
	3 mo	15 mo	27 mo	3 mo	15 mo	27 mo	Geno	Age	Geno*Age
Body Weight	25.75±0.56^a^	31.75±0.76^b^	29.78±0.80^b^ *	26.4±0.71^a^	33.1±0.29^b^	33.03±0.87^b^ *	0.015	<0.0001	0.194
Lean Body Mass	19.53±0.35^a^	25.74±0.51^b^	24.98±0.67^b^ *	21.55±0.58^a^	27.94±0.30^b^	27.62±0.79^b^ *	0.0002	<0.0001	0.862
Liver	1.19±0.067^a^	1.44±0.052^b^	1.48±0.101^b^	1.26±0.050^a^	1.67±0.095^b^	1.59±0.075^b^	0.070	0.0014	0.070
Spleen	0.062±0.005	0.088±0.007	0.136±0.059	0.060±0.005	0.075±0.002	0.136±0.006	0.437	0.465	0.674
Kidneys	0.328±0.016^a^	0.483±0.025^b^	0.435±0.022^b^	0.336±0.008^a^	0.515±0.006^b^	0.466±0.020^b^	0.227	<0.0001	0.846
Lungs	0.144±0.012^a^	0.175±0.006^b^	0.181±0.013^b^	0.154±0.012^a^	0.190±0.003^b^	0.192±0.010^b^	0.254	0.008	0.977
Heart	0.130±0.010^a^	0.165±0.008^b^	0.167±0.004^b^	0.136±0.008^a^	0.164±0.009^b^	0.167±0.005^b^	0.789	<0.0001	0.876
Brain	0.386±0.025^a^	0.442±0.007^b^	0.447±0.007^b^	0.390±0.010^a^	0.434±0.023^b^	0.451±0.005^b^	0.990	<0.0001	0.876
Crude Organ Weight^2^	2.03±0.101^a^	2.53±0.063^b^	2.53±0.063^b^	2.12±0.046^a^	2.79±0.077^b^	2.68±0.095^b^	0.071	<0.0001	0.772

1 Data are presented as means ± SEM; superscript letters that differ indicate differences between ages within genotype, Bonferroni corrected *P* value <0.05;

2 Crude organ weight is the sum of liver, spleen, kidney, lung, heart and brain weights.

* indicates difference within age between genotype, Bonferroni corrected *P* value <0.05.

### Respiratory Quotient

#### Shc proteins and Respiratory Quotient

**Figure 3:**
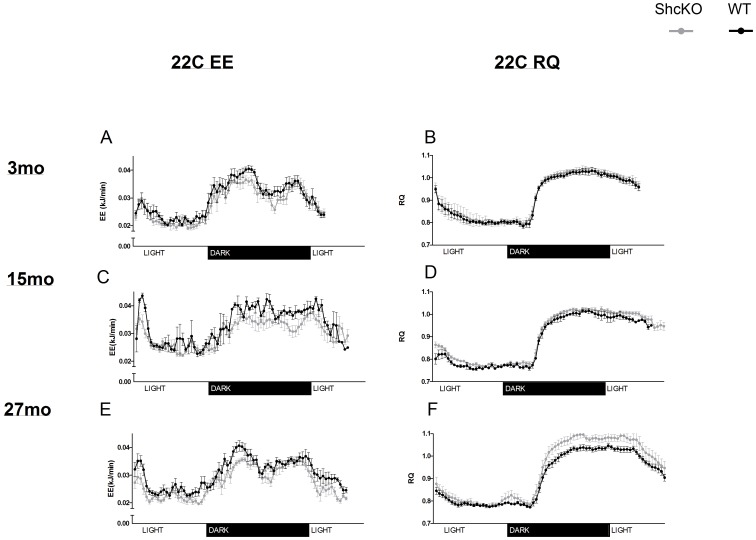
Energy expenditure (EE) and respiratory quotient (RQ) in ShcKO and wild-type (WT) mice housed at 22°C. Mean (±SEM) EE and RQ values collected over a 24 hour period in 3 mo (A&B), 15 mo (C&D), and 27 mo (E&F) mice. ShcKO = p66 Shc(−/−) mice, mo = months.

**Figure 4:**
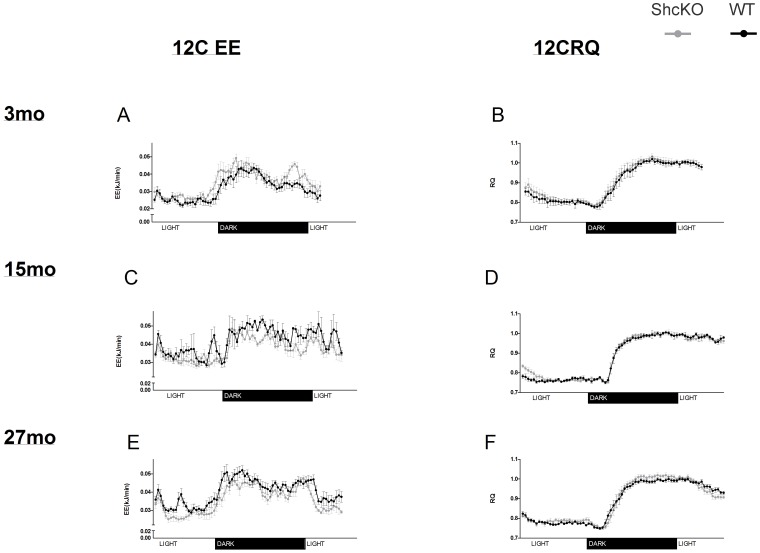
**Energy expenditure (EE) and respiratory quotient (RQ) in ShcKO and wild-type (WT) mice housed at 12°C.** Mean (±SEM) EE and RQ values collected over a 24 hour period in 3 mo (A&B), 15 mo (C&D), and 27 mo (E&F) mice. ShcKO = p66 Shc(−/−) mice, mo = months.

^α, ¥, $,+^ values are presented as least square mean ± SEM, adjusted for MEI, BW, FFM, and ORGAN, respectively.

Table 2 and figures 3 and 4 provide detailed RQ data under 22°C and 12°C conditions in terms of 24h average RQ (Table 2), RQ plotted against time (Figures 3 and 4) and data partitioned by fed and fasting conditions (Table 3 and 4). Under both fed and fasted conditions, there were no significant differences between genotypes in pattern of change in RQ with aging or cold exposure. Thus, insignificant interactions were systematically dropped from the final model through stepwise backward elimination process. Both genotypes showed a decrease (P<0.01 for all ages) in RQ with cold exposure.

**Table 2:** 24 hour energy expenditure (EE) and respiratory quotient (RQ) in ShcKO and wild-type (WT) mice housed at 22°C or 12°C.^1^

	P66 Shc(−/−)			WT			*P* Value		
	3 mo	15 mo	27 mo	3 mo	15 mo	27 mo	Geno	Age	Geno*Age
**22°C**									
RQ	0.921±0.011	0.904±0.004	0.939±0.015	0.917±0.008	0.883±0.004	0.906±0.006	0.050	0.067	0.912
RQ_MEI_^α^	0.919±0.010	0.908±0.013	0.938±0.010	0.914±0.007	0.885±0.012	0.909±0.007	0.020	0.051	0.912
EE (kJ/mouse)	40.072±0.664^a^	43.475±1.398^b^	39.406±0.922^a^	42.722±0.993^a^	47.943±1.108^b^	43.846±1.465^a^	0.0003	0.0047	0.685
EE_BW_ (kJ)^¥^	42.173±1.001^a^	41.218±1.194^a^	38.577±0.812^b^	47.024±1.174^a^	44.240±1.606^a^	40.301±1.094^b^	0.012	0.0001	0.929
EE_FFM_ (kJ)^$^	42.822±1.409^a^	41.277±1.374^a^	37.816±1.064^b^	45.997±1.566^a^	44.979±2.121^a^	41.195±1.434^b^	0.115	0.0009	0.822
EE_ORGAN_ (kJ)^+^	42.439±1.413^a^	42.848±1.072^a^	38.782±0.890^b^	46.593±1.833^a^	45.506±1.806^a^	42.451±1.041^b^	0.006	0.002	0.495
**12°C**									
RQ	0.907±0.013	0.886±0.003	0.888±0.007	0.897±0.009	0.882±0.003	0.883±0.006	0.412	0.077	0.506
RQ_MEI_^α^	0.908±0.009^a^	0.883±0.010^b^	0.888±0.008^ab^	0.896±0.005^a^	0.877±0.009^b^	0.887±0.005^ab^	0.173	0.039	0.687
EE (kJ/mouse)	49.866±0.897^a^	53.558±1.523^b^	51.347±1.163^ab^	53.431±1.769^a^	60.262±1.688^b^	57.547±1.916^ab^	0.0002	0.0195	0.311
EE_BW_ (kJ)^¥^	51.004±1.513	52.338±1.695	51.149±1.091	58.531±2.106	56.689±2.598	54.204±1.694	0.005	0.595	0.098
EE_FFM_ (kJ)^$^	52.518±2.019	51.826±1.691	50.144±1.297	60.574±2.363	55.066±2.653	52.929±2.653	0.076	0.064	0.284
EE_ORGAN_ (kJ)^+^	51.472±2.044	53.511±1.412	51.300±1.166	59.046±2.327	56.521±2.068	55.592±1.175	0.009	0.498	0.208

MEI, metabolizable energy intake; BW, body weight; FFM, fat-free mass; ORGAN, crude organ mass (sum of liver, kidney, heart, and brain mass); EE is expressed as EE kJ/mouse (kJ per mouse) and EE_BW, FFM, ORGAN_ (kJ normalized by BW, FFM, and ORGAN).

1 Data are presented as means ± SEM unless otherwise indicated; superscript letters that differ indicate differences between ages within temperature and genotype, Bonferroni corrected *P* values provided in table;

Because MEI had a significant effect on RQ (P<0.05), we utilized this measure of food intake as a covariate when analyzing RQ data. In the fed state at 22°C, ShcKO animals demonstrated higher RQ and RQ_MEI_ values (P<0.05) than that of WT animals. These results are consistent with an increase in glucose utilization in the ShcKO compared to WT mice following feeding. However, we did not find a significant genotype effect on either RQ or RQ_MEI_ in the fasted state at 22°C. Furthermore, there was not a significant genotype effect on RQ or RQ_MEI_ at 12°C in either the fed or fasted state.

**Table 3:** Energy expenditure and respiratory quotient in ShcKO and wild-type (WT) mice housed at 22°C.^1^

	ShcKO			WT			*P* Value		
	3 mo	15 mo	27 mo	3 mo	15 mo	27 mo	Geno	Age	Geno*Age
**Fasted**									
RQ	0.831±0.018^a^	0.796±0.0030^b^	0.808±0.011^b^	0.0823±0.009^a^	0.779±0.0053^b^	0.793±0.0053^b^	0.180	0.005	0.921
RQ_MEI_^α^	0.829±0.0122^a^	0.798±0.014^b^	0.808±0.0115^b^	0.819±0.0078^a^	0.779±0.0126^b^	0.796±0.0074^b^	0.179	0.046	0.931
EE (kJ/min/mouse)	0.0241±0.0005^a^	0.0267±0.00045^b^	0.0240±0.00091^ab^	0.026±0.00011^a^	0.028±0.0011^b^	0.027±0.00090^ab^	0.002	0.032	0.728
EE_BW_ (kJ/min)^¥,^	0.0259±8.42E-4^a^	0.0251±8.70E-4^ab^	0.0235±5.93E-4^b^	0.0290±1.27E-4^a^	0.0261±1.44E-3^ab^	0.0247±9.89E-4^b^	0.068	0.004	0.873
EE_FFM_ (kJ/min)^$^	0.027±9.52E-4^a^	0.024±7.97E-4^ab^	0.022±6.11E-4^b^	0.028±1.58E-3^a^	0.026±1.77E-3^ab^	0.025±1.20E-3^b^	0.341	0.0002	0.888
EE_ORGAN_ (kJ/min)^+^	0.0261±1.18E-3^a^	0.262±5.98E-4^ab^	0.236±5.88E-4^a^	0.028±1.99E-3^a^	0.027±1.04E-3^ab^	0.026±8.59E-4^a^	0.021	0.033	0.639
**Fed**									
RQ	1.016±0.011^a^	0.998±0.0068^a^	1.051±0.019^b^	1.006±0.010^a^	0.979±0.0053^a^	1.002±0.00935^b^	0.016	0.040	0.289
RQ_MEI_^α^	1.01±0.013^a^	1.00±0.0159^a^	1.05±0.013^b^	0.999±0.009^a^	0.981±0.014^a^	1.01±0.008^b^	0.010	0.009	0.506
EE (kJ/min/mouse)	0.0316±0.0012^a^	0.0331±0.0016^b^	0.0301±0.005^a^	0.0340±0.0013^a^	0.0375±0.00067^b^	0.0333±0.0011^a^	0.002	0.025	0.549
EE_BW_ (kJ/min)^¥,^	0.033±1.42E-3^a^	0.031±1.46E-3^a^	0.029±1.00E-3^b^	0.037±9.61E-4^a^	0.034±1.09E-3^a^	0.030±7.47E-4^b^	0.064	0.0005	0.453
EE_FFM_ (kJ/min)^$^	0.032±2.21E-3^a^	0.033±2.48E-3^a^	0.030±1.26E-3^b^	0.037±1.34E-3^a^	0.035±1.51E-3^a^	0.031±1.02E-3^b^	0.155	0.004	0.618
EE_ORGAN_ (kJ/min)^+^	0.033±2.25E-3^a^	0.033±1.59E-3^a^	0.030±5.98E-4^b^	0.037±1.31E-3^a^	0.035±9.13E-4^a^	0.032±7.96E-4^b^	0.035	0.006	0.690

MEI, metabolizable energy intake; BW, body weight; FFM, fat-free mass; ORGAN, crude organ mass (sum of liver, kidney, heart, and brain mass); EE is expressed as EE kJ/min/mouse (kJ per min per mouse) and EE_BW, FFM, ORGAN_ (kJ/min normalized by BW, FFM, and ORGAN).

1 Data are presented as means ± SEM unless otherwise indicated; superscript letters that differ indicate differences between ages within temperature and feed period, Bonferroni corrected *P* values provided in table;

α, ¥, $,+ values are presented as least square mean ± SEM, adjusted for MEI, BW, FFM, and ORGAN, respectively.

When looking at 24h overall RQ at 22°C, ShcKO mice maintained a significantly higher RQ (*P* = 0.050) and RQ_MEI_ (P = 0.020) than WT mice, and this was entirely due to increased fed state RQ values in the ShcKO animals.

**Table 4:** Energy expenditure and respiratory quotient in ShcKO and wild-type (WT) mice housed at 12°C.^1^

	P66 Shc(−/−)			WT			*P* Value		
	3 mo	15 mo	27 mo	3 mo	15 mo	27 mo	Geno	Age	Geno*Age
**Fasted**									
RQ	0.824±0.015^ a^	0.776±0.0054^b^	0.785±0.007^b^	0.0813±0.013^a^	0.769±0.0032^b^	0.782±0.0045^b^	0.420	0.0002	0.922
RQ_MEI_^α^	0.826±0.0107^a^	0.774±0.0124^b^	0.787±0.0101^b^	0.811±0.0082^a^	0.764±0.0135^b^	0.7828±0.0077^b^	0.281	0.0001	0.838
EE (kJ/min/mouse)	0.0308±0.00079	0.0339±0.0012	0.0321±0.0014	0.0340±0.0013	0.0367±0.0017	0.369±0.00153	0.001	0.104	0.685
EE_BW_ (kJ/min)^ ¥,^	0.0315±1.57E-3	0.0332±1.76E-3	0.032±1.13E-4	0.0370±0.0019	0.0346±2.36E-3	0.0349±1.54E-3	0.022	0.927	0.958
EE_FFM_ (kJ/min)^$^	0.033±2.06E-3	0.032±1.17E-3	0.031±1.32E-3	0.033±2.06E-3	0.032±1.73E-3	0.031±1.32E-3	0.150	0.289	0.856
EE_ORGAN_ (kJ/min)^+^	0.0299±1.11E-3^a^	0.0341±1.19E-3^a^	0.032±1.14E-3^a^	0.036±2.18E-3^a^	0.034±2.09E-3^a^	0.035±1.32E-3^a^	0.026	0.699	0.614
**Fed**									
RQ	0.993±0.014	0.979±0.0042	0.980±0.0085	0.986±0.011	0.979±0.0065	0.972±0.0089	0.491	0.361	0.960
RQ_MEI_^α^	0.997±0.010	0.977±0.011	0.983±0.009	0.982±0.009	0.971±0.014	0.974±0.008	0.219	0.284	0.918
EE (kJ/min/mouse)	0.0386±0.0013	0.0399±0.0012	0.0388±0.000703	0.0403±0.0013	0.0462±0.00078	0.0427±0.0013	0.001	0.086	0.311
EE_BW_ (kJ/min)^¥,^	0.039±1.39E-3	0.039±1.56E-3	0.038±1.00E-4	0.044±1.33E-3	0.043±1.64E-3	0.040±1.07E-3	0.028	0.343	0.236
EE_FFM_ (kJ/min)^$^	0.039±1.93E03	0.039±1.62E-3	0.038±1.24E-3	0.045±1.46E-3^a^	0.042±1.64E-3^a^	0.039±1.11E-3^b^	0.170	0.074	0.298
EE_ORGAN_ (kJ/min)^+^	0.041±1.31E-3^a^	0.040±1.20E-3^a^	0.039±7.60E-4^a^	0.045±1.65E-3^a^	0.043±8.15E-4^a^	0.041±6.69E-4^a^	0.035	0.255	0.061

MEI, metabolizable energy intake; BW, body weight; FFM, fat-free mass; ORGAN, crude organ mass (sum of liver, kidney, heart, and brain mass); EE is expressed as EE kJ/min/mouse (kJ per min per mouse) and EE_BW, FFM, ORGAN_ (kJ/min normalized by BW, FFM, and ORGAN).

1 Data are presented as means ± SEM unless otherwise indicated; superscript letters that differ indicate differences between ages within temperature and feed period, Bonferroni corrected *P* values provided in table;

α, ¥, $,+ values are presented as least square mean ± SEM, adjusted for MEI, BW, FFM, and ORGAN, respectively.

#### Age and Respiratory Quotient

There was a clear trend towards an age-related change in 24 h RQ and RQ_MEI_ at 22°C, with the 15 mo old animals showing the lowest RQ values (*P* = 0.051 to 0.077) (Table 2). At 12°C, there was a decrease (P<0.05) in RQ_MEI_ from 3 to 15 months of age.

In the fed state, all animals had RQ values near 1.0, indicating heavy reliance on glucose as an energy substrate. With a 12 hour fast, all mice showed average RQ values near 0.8, consistent with a shift toward increased reliance on fatty acids as energy substrates. There were small, but significant, changes in RQ and RQ_MEI_ with aging in both the fed and fasted states. At 22°C, there was an increase (P<0.05) in fed state RQ and RQ_MEI_ in the 27 mo old mice compared to the other age groups. However, at 12°C, there were no differences between age groups in fed state RQ and RQ_MEI_. With fasting, there was an increase in RQ and RQ_MEI_ in the 3 mo old mice compared to other age groups at both 12 and 22°C.

### Energy Expenditure

Under both fed and fasted conditions and regardless of how EE was normalized, the pattern of change in EE in response to age and temperature was not different between genotypes. Thus, these insignificant interactions were removed from the final model through stepwise backward elimination process. Tables 2, 3 and 4 and Figures 3 and 4 provide detailed EE data under 22°C and 12°C conditions in terms of 24 h total EE (Table 2), EE plotted against time and data partitioned by fed and fasting conditions (Tables 3 and 4).

### Shc Proteins and Energy Expenditure

At both 22°C and 12°C, ShcKO mice demonstrated significantly lower 24 h EE than WT mice (P<0.01) when EE was expressed as either kJ per mouse, or normalized by either BW or ORGAN (Table 2). Decreases in both fed and fasted EE expressed as kJ/min/mouse or normalized by ORGAN contributed to the observed decrease in 24 hour EE in the ShcKO compared to WT mice. Furthermore, EE adjusted for BW demonstrated a trend of lower EE in ShcKO mice than that of WT mice (P = 0.068 and 0.064 for fasted and fed, respectively). At 12°C both fasted and fed EE was decreased compared to WT animals in the ShcKO mice when expressed as kJ per mouse or adjusted for either BW or organ mass. (*P* = 0.022 and 0.028 for fasted and fed, respectively). In this study, WT mice demonstrated higher FFM than that of ShcKO mice and when EE was adjusted for this variable under 22°C and 12°C conditions, EE was no longer significantly lower in ShcKO mice, regardless of temperature or whether animals were fasted or fed.

### Age and Energy Expenditure

Under 22°C conditions, age had a significant effect on total 24 h EE regardless of how EE data was normalized (P<0.005) (Table 2). There was an increase in EE (kJ/mouse) at 15 months compared to either 3 or 27 months of age. When EE was normalized for BW, FFM or organ weight there was a decrease in EE in the 27 month old mice compared to both the 3 and 15 mo groups. The effect of age was significant under 12°C conditions when EE data was expressed as kJ/mouse (Table 2). However, there were no significant differences (P>0.05) between age groups under 12°C conditions when EE was normalized by BW, FFM, or ORGAN (P>0.05).

There was a significant age effect on EE under fasted and fed conditions at 22°C (Table 3), regardless of how EE data was expressed (P<0.05). This age effect on EE followed a pattern of an increase in EE from age 3 to 15 mo, followed by a decrease in EE at 27 mo which, decreased below that of 3 mo of age. Because this pattern occurred regardless of how EE data was adjusted, it indicates an age-related decline in whole body EE, independent of changes in BW or FFM. These results indicate that both fasted and fed EE contribute to the age-related decrease in whole body 24 hour EE.

The ratio of dark EE to light EE was used to indicate the magnitude of diurnal changes in EE with aging or cold exposure. Since physical activity is a major contributor to diurnal changes in EE, these measures may also provide an indication of physical activity. Because there were no differences between the two genotypes in the pattern of change in the ratio of dark to light EE in response to aging or cold exposure, these insignificant interaction terms were systematically removed from the final model through stepwise backward elimination process. Neither genotype nor age had a significant impact on the ratio of dark to light EE. However, there was a decrease (P<0.002) in the ratio of dark to light EE in both genotypes at 12°C compared to 22°C (Table 5).

**Table 5:** Ratio of dark to light energy expenditure in ShcKO and wild-type (WT) mice housed at 22°C or 12°C.^1^

	ShcKO			WT			*P* Value		
	3 mo	15 mo	27 mo	3 mo	15 mo	27 mo	Geno	Age	Geno*Age
**D:L Ratio**									
**22°C**	1.31±0.06	1.24±0.05	1.26±0.04	1.29±0.05	1.31±0.03	1.23±0.01	0.814	0.416	0.600
**12°C**	1.25±0.06	1.17±0.03	1.22±0.05	1.18±0.03	1.26±0.04	1.16±0.03	0.349	0.818	0.352

1 Data are presented as means ± SEM.

## Discussion

### Shc Proteins and Body Composition

It has previously been shown that body composition is altered in ShcKO compared to WT mice [4], [8]. In particular, it was reported that body weight is significantly decreased in young (2 mo) ShcKO mice compared to that of WT controls, and this decrease in body weight was due to lower fat pad weights in ShcKO mice compared to wild-type mice [8]. In addition, it has been shown that body, fat pad, and liver weights are significantly lower in young (3–5 month old) ad libitum fed ShcKO compared to WT mice [4]. However, it is important to note that these studies were all completed in young, adult animals and little is known about the body composition changes in older ShcKO mice. The present study showed that ShcKO animals demonstrate a slightly lighter total body mass and fat free mass compared to that of WT animals. However, this difference was only significant at 27 mo of age, suggesting that the influence of Shc proteins on total body mass and lean mass may be magnified at older age. To our knowledge, our study is the first to investigate the impact of Shc proteins on body mass and body composition in older mice. While previous studies have reported that Shc proteins influence fat pad weights [4], [8], the results of the present study indicates that Shc proteins also alters lean mass, at least in older mice.

### Age and Body Composition

It has been well documented that changes in body composition occur with aging in humans [31], [32] as well as mice [33]. Percent body fat increases with age and lean mass decreases with age in humans [34]. It has also been reported that body and lean mass are decreased with age in male C57BL/6-aa mice (with aa denoting homozygosity for the non-agouti or black coat color in this study) [33]. However, a study in C57BL/6JOlaHsd mice did not show a significant decrease in body mass from 11 to 27 mo of age in cross-sectional animals used for body composition measurements [35]. The authors also reported an increase in lean body mass from 3 to 11 months of age but no decrease in lean mass from 11 mo to 19 mo and 19 mo to 27 mo [35]. Similarly, the results of our study show an age related increase in both body mass and lean mass from 3 to 15 mo of age but no significant decrease in body mass and lean mass from 15 mo to 27 mo of age. The findings of Vaanholt et al. also demonstrated an age-related increase in organ mass among all organs with the exception of liver and brain [35]. However, we found that all organs, with the exception of spleen, increased in weight from 3 to 15 months of age with no further increase in weight from 15 to 27 months of age (Table 1). The reason for the differences in age-related changes in organ weights between studies are not entirely clear although diet and differences in source of C57BL/6 mice may be contributing factors. Based on patterns of weight gain as documented by growth curves for C57BL/6 mice, we would expect the pattern of weight gain observed in our study [36]. Furthermore, also based on these growth curves, we would expect weight loss after 20 mo of age. However, a significant decrease in body weight was not observed in the present study from 15 to 27 months of age, and this likely reflects the fact that only mice that were apparently healthy were included in the 27 month group. Thus, weight in the oldest group was not influenced by animals exhibiting signs of age-related disease. The present study finds little evidence of substantial decreases in body or organ weight from middle age (15 mo.) to advanced age (27 mo.) in healthy male C57BL/6 mice. It is also important to note that this finding may be due, in part, to the small number of animals in the 15 month group.

### Shc Proteins and Energy Expenditure

When looking at the effect of Shc proteins on EE, we found a decrease in EE expressed as kJ/mouse or normalized for body weight or organ mass in the ShcKO compared to WT mice at 22°C when examined across age-groups. This result differs from a previous study which found that oxygen consumption (ml/g/hr) was significantly increased in male ShcKO compared to WT mice [8]. There are at least two possible reasons for the differences in EE between studies. First, little information is provided in the Bernakovich et al. [8] study about the calorimetry measurements. Thus, it is possible that differences in adaptation time or calorimeter environment could contribute to the differences in results between studies. Second, the method of normalizing EE for body size and composition could contribute to the differences between studies. In the present study, the method of normalizing EE data had a major influence on whether differences were observed between groups of mice. Expressing EE per unit of body mass as a ratio has been criticized and ANCOVA with body weight or a measure of body composition as a covariate is the most appropriate method to compare EE data [37]–[39]. It is important to note that the decrease in EE (kJ/mouse) in the ShcKO animals is small (a decrease of less than 10% compared to WT). This is likely why we did not see genotype differences within each age group. It is likely that a relatively large sample size is needed to detect the small changes in EE between genotypes.

At first glance, the decrease in EE (kJ/mouse) in the ShcKO compared to WT mice does not appear to be consistent with the decreased body weight in 27 mo ShcKO compared to WT mice and the lack of difference in food intake between genotypes. There are a couple of possible reasons for this difference in body weight. First, it should be noted that food intake was measured under the same conditions as EE (cages with the same dimensions as the home cage and bedding) and it can be difficult to detect small differences in food intake when these measurements require sifting of bedding and collecting food remaining in the cage. It is likely that the EE measurements are able to detect smaller changes than the food intake measurements. Second, it is possible that age-related changes in energy digestion are different between ShcKO and wild-type mice. Future studies should investigate the influence of Shc proteins on digestible energy in older animals.

Since FFM, BW, and ORGAN are major contributors to EE [37], [40], [41], it is important to take into consideration differences in these variables when determining how Shc proteins and aging may influence whole body energy expenditure. It should be pointed out that WT mice demonstrated higher FFM than that of ShcKO mice and when EE was adjusted for this variable under 22°C and 12°C conditions, EE was no longer significantly lower in ShcKO mice, regardless of whether animals were fasted or fed. Thus, FFM is a major factor contributing to differences in EE (kJ/mouse) between genotypes. The observation that ShcKO showed lower rates of EE than WT animals (*P*≤0.05) when EE was normalized by ORGAN under both fed and fasted conditions at 22°C is important and brings to question whether crude organ mass or total fat free mass is the most appropriate factor for normalizing energy expenditure. This question is the subject of debate in human [40], [42]–[45] and rodent [39], [46], [47] studies. Our study provides a further example that the covariant used to normalize EE data can have a major impact on the conclusion about the influence of genotype or treatment on EE. For example, in this study if we were to rely entirely on FFM, genotype-related changes in EE adjusted for ORGAN or BW would be overlooked. The internal organs are responsible for greater than 60% of resting energy expenditure, despite the fact that they account for less than 10% of body weight [48]. Thus, it is possible that EE_ORGAN_ may provide a better indication of resting energy expenditure than EE_FFM_, which largely reflects the muscle mass.

The mechanism responsible for the decrease in EE in the ShcKO is not entirely known. It has previously been reported that knockout of p66 Shc increases mitochondrial uncoupling and oxygen consumption in brown adipose tissue [9]. In contrast, it has also been reported that p66 Shc localizes to mitochondria and increases oxygen consumption in mouse embryonic fibroblasts [12]. The influence of Shc proteins on oxygen consumption/energy expenditure in other tissues is not known. However, the results of the present study are consistent with the idea Shc proteins may stimulate a net increase in mitochondrial oxygen consumption.

When looking at the degree to which EE was increased in ShcKO mice in response to cold, we found that the magnitude of the EE increase in these mice was similar to that observed in WT animals (within 3 to 7%, depending on the method of EE normalization). The impact of cold stress on p66 Shc(−/−) mice has been previously reported [8], [49]. In contrast to the study design of the experiments presented here, the aforementioned studies exposed mice acutely for 6 h to 5°C [8] and chronically for 3 h per day to 4°C [49]. Both of these studies reported a faster drop in body temperature in p66 Shc(−/−) mice compared to that of WT animals in repose to cold exposure. Furthermore, the study of Giorgio et al. [49], reported that chronic cold exposure resulted in a significant decrease in body weight in p66 Shc(−/−) but not WT mice. The current study used a more moderate cold stress (12°C compared to 4–5°C) and we exposed our animals for a period of 24 continuous hours compared to shorter periods of time implemented in the previous studies. Our study indicates that ShcKO mice do not demonstrate an impairment in the ability to increase EE in response to 24 hour moderate cold stress.

### Shc Proteins and Substrate Oxidation

In the fed state under 22°C conditions, ShcKO animals had higher RQ and RQ_MEI_ values than that of WT animals. It is important to note that genotype differences observed in this study were small in magnitude and only significant when analyzed across age groups. Furthermore, these differences were driven entirely by RQ in the fed state, since there were no differences between genotype in fasting RQ. Increased insulin sensitivity and glucose tolerance in p66 Shc(−/−) mice has been reported [4], [9] and such differences in insulin sensitivity and glucose oxidation would be expected to be seen after a meal. Thus, the higher fed state RQ values in the ShcKO compared to WT are consistent with the idea of increased insulin sensitivity in the ShcKO animals.

### Age and Energy Expenditure

Similar to previously reported findings that aging induces a decrease in whole body energy expenditure in humans independent of changes in body composition [50], [51], we found a significant age effect on EE under fasted and fed conditions at 22°C (Table 3). Because this pattern occurs regardless of how EE data was adjusted, it indicates a decline in whole body EE with advanced age in mice, independent of changes in BW or FFM. Few studies have investigated the impact of aging on energy expenditure in a rodent model. A study in Fischer 344 rats found no effect of age on EE [52]. However the oldest age group in this study was 24 mo and rats may respond energetically in a manner different from that of mice. Additionally, a study investigating the impact of age and mouse strain on energy expenditure found no significant age related changes in EE in 6 versus 23 mo C57B/6 mice [47]. It is possible that older ages are needed to see age-related changes in EE in these mice. Nonetheless, the results of the present study indicate that EE adjusted for BW, FFM or ORGAN is decreased in 27 mo old C57B/6 mice compared to younger (3 or 15 mo) animals.

Age did not impact the animals’ ability to increase EE in response to a shift from 22°C to 12°C conditions. In fact, 27 mo mice demonstrated a similar magnitude of increase in 24 h total EE_BW_ in response to cold as 3 mo old animals. Such magnitude of change in EE in response to cold in all animals remained consistent, regardless of how EE data was expressed (Table 2). This observation that age did not impair a cold-induced increase in whole body energy expenditure may also explain why there was no clear age effect on EE under 12°C conditions when EE was normalized for BW, FFM, or ORGAN (Table 2). Studies in C57BL/6J mice have reported an age-related decline in cold-induced increase in heat production [14]–[16]. However, these studies involved restrained animals exposed to 6°C for a period 6 hours. The findings of the present study indicate that older mice can increase EE to a level comparable to that of younger animals when exposed to 12°C for 24 hours.

### Age and Substrate Oxidation

An age-related decline in the capacity for lipid oxidation has been reported in a mouse model of accelerated aging [53] and human studies [54], [55]. Consistent with these findings, our observation of an age-related increase in 22°C RQ and RQ adjusted for MEI under both fed and fasted states from 18 to 27 mo of age suggests that age, does in fact, impact substrate oxidation at the whole-animal level in a mouse model. However, our findings that under 12°C conditions, fasting RQ and RQ adjusted for MEI decreased significantly from 3 mo to 18 and 27 mo of age suggests that the capacity for lipid oxidation is not impaired at these ages under metabolically demanding conditions, such as cold exposure.

### Shc Proteins

It is not possible at this time to determine specifically which Shc protein is responsible for the changes in energy expenditure and substrate oxidation observed in the ShcKO mice. It has previously been shown that the ShcKO mice show a complete absence of p66 Shc in all tissues and decreased levels of p46 Shc and p52 Shc in muscle, liver and other tissues [4]. Additional studies are needed to determine which specific Shc isoforms influence whole animal energy expenditure and substrate oxidation. Additionally, because only male mice were used in the present study, it will be of interest for future studies to investigate how sex differences may play a role in the influence of Shc proteins on body composition and whole body energy metabolism.

### Conclusion

In conclusion, our results inidicate in C57BL/6J mice that aging is associated with a significant decrease in whole body energy expenditure, independent of changes in lean mass. Thus, these mice appear to model age-related changes in EE in humans. This study also shows that deletion of Shc proteins alters EE and RQ. Specifically, EE is decreased in ShcKO compared to WT mice when expressed per mouse or adjusted for BW or crude organ mass. However, Shc proteins do not affect age-related or cold induced changes in EE or RQ. Additionally, Shc proteins impact whole body substrate utilization under fed conditions and this data is consistent with previous findings of enhanced insulin sensitivity in p66Shc(−/−) mice. Thus, Shc proteins should be considered as contributing factors to whole body energy metabolism.
